# The urgent need for patients’ diagnoses and outcome feedback in Germany’s emergency medical services — insights from a web-based survey

**DOI:** 10.1186/s12873-025-01218-8

**Published:** 2025-04-20

**Authors:** Anika Kästner, Petra Lücker, Lutz Fischer, Timm Laslo, Berthold Henkel, Jennifer Ehleben, Wolfgang Hoffmann, Neeltje van den Berg

**Affiliations:** 1 Institute for Community Medicine, Section Epidemiology of Health Care and Community Health, University Medicine Greifswald, Ellernholzstraße 1-2, 17487 Greifswald, Germany; 2 Physical and Rehabilitation Medicine, Clinic and Polyclinic for Orthopedics, Trauma Surgery and Rehabilitation Medicine, University Medicine Greifswald, Greifswald, Germany; 3 Communal Rescue Services, District of Vorpommern-Greifswald, Greifswald, Germany; 4 Department of Anesthesiology, University Medicine of Greifswald, Greifswald, Germany

**Keywords:** Emergency medical services, Survey, Feedback, Digital feedback systems, Prehospital care

## Abstract

**Background:**

German Emergency Medical Services (EMS) face growing scrutiny due to regional disparities in quality of care. It is unclear if and how feedback in general is currently provided to EMS staff in Germany, and whether EMS staff receives feedback on patients’ diagnoses and outcomes.

**Methods:**

A web-based survey was conducted from June to August 2024 among physician and non-physician EMS staff, focusing on current feedback reception and the perceived need for feedback systems.

**Results:**

A total of *N* = 428 EMS professionals participated in the survey. One-third of the participants reported receiving no feedback (*n* = 136, 31.8%), while over half of those who did, received feedback infrequently (*n* = 157, 55.5%). Informal feedback was the main source, with 95.4% of respondents desiring official feedback on the confirmed in-hospital diagnosis, e.g., to learn from previous cases. While 57.5% of emergency physicians occasionally or frequently receive information about the further course of treatment for patients after transport to the hospital, this was reported by only 14.3% (advanced emergency medical technicians) to 29.2% (emergency medical technicians) of non-physician EMS staff. More than 85% of the respondents stated that diagnosis feedback would improve the quality of EMS.

**Conclusion:**

Structured feedback mechanisms, essential for quality assurance and improvement, are largely absent for EMS staff in Germany, especially for non-physicians. A strong desire among EMS staff for structured feedback on patients’ diagnoses and outcomes was found, which could improve quality of care and staff competence development. However, significant infrastructural and legal barriers persist, hindering the implementation of standardized digital feedback systems within Germany’s federalized EMS structure.

**Supplementary information:**

The online version contains supplementary material available at 10.1186/s12873-025-01218-8.

## Introduction

Emergency Medical Services (EMS) provide critical, life-saving care and are an integral part of the healthcare system, forming a key component of the German critical infrastructure. However, the EMS in Germany is under growing pressure. Emergency deployments have been increasing by approximately 5% annually, a rise that cannot be explained solely by more low-acuity cases [1, 2]. This trend further intensifies the already excessive workload and existing staff shortages. A recent investigation by the German broadcaster *Südwestrundfunk* revealed disparities in EMS quality, particularly in response times to sudden cardiac arrest—only 24 out of 283 EMS regions met the target of an eight-minute arrival in 80% of cases [3].

A nationwide law to reform emergency care is currently progressing through the legislative process. Its objective is to improve the EMS efficiency and ensure that emergency services are tailored to patient needs by directing them to the most appropriate healthcare provider. Furthermore, this reform intends to reduce the burden on emergency and ambulance services, optimizing resource allocation and improving patient outcomes. In response to the growing staff shortage in German EMS, paramedics’ competencies are being expanded, and they are being given more responsibility, in addition to the successful deployment of tele-emergency physicians [4–6]. For instance, since the introduction of a telemedicine system in Mecklenburg-Western Pomerania, the percentage of emergency calls requiring additional on-site emergency physician assistance has decreased from around 55% to approximately 38% [7]. The responsibility for quality assurance lies with each federal state.

A retrospective analysis of over 1000 patients treated by emergency physicians in Brandenburg, Germany found that more than 20% of the pre-hospital suspected diagnoses made by emergency physicians differed from the hospital discharge diagnosis [8]. Notably, for cases presenting with dyspnea as a primary symptom, there was a high discrepancy in diagnostic accuracy, with correct suspected diagnoses of pneumonia at 32% (17 out of 53) and cardiac decompensation at 53% (50 out of 94) [9]. As noted by the authors, ‘dyspnea’ remains a prehospital challenge due to its diverse underlying causes. Consequently, the need for a suitable quality management and assurance system, for both physician and non-physician staff, is further increasing [10]. Feedback is essential for quality assurance, allowing performance to be evaluated and areas for improvement to be identified [11–13].

A systematic review by Rogers et al. examined whether audit and feedback interventions affected the emergency physician performance and found that 23 of the 24 studies included reported performance improvements [14]. Another systematic review and meta-analysis examined the types and effects of feedback for EMS staff, concluding that feedback may improve both staff performance and patient care [15]. However, effects of patient outcome feedback have been rarely studied so far. Feedback on patients’ diagnoses and outcomes following EMS intervention is the most difficult to provide, as it usually requires information on the inpatient treatment course of patients, which is difficult to obtain due to the many different service providers involved in patient care [16].

It is unclear if and how feedback is currently provided to EMS personnel in Germany, and whether EMS personnel receive feedback on patients’ diagnoses and outcomes in general. Therefore, a web-based survey was conducted among physician and non-physician EMS personnel to examine whether at all and, if so, to what extent they currently receive feedback and whether they consider a diagnosis feedback system in the EMS to be necessary for quality assurance measures.

## Methods

In collaboration with the Communal Rescue Services of the District Vorpommern-Greifswald in the Northeast of Germany, a web-based anonymous survey was conducted from June 17 to August 31, 2024 via SoSci Survey [17] among emergency ambulance personnel in Germany with a focus on Mecklenburg-Western Pomerania. Eligibility criteria for study participation included (a) consent to the anonymized use of data for research purposes, and (b) employment in the EMS sector including emergency physicians, paramedics (German: Notfallsanitäter), emergency medical technicians (EMT; German: Rettungssanitäter), advanced emergency medical technicians (AEMT; German: Rettungsassistent), emergency medical responders (EMR; German: Rettungshelfer), and trainees in these professions.

### Development and description of the questionnaire

The questionnaire was developed based on a comprehensive literature review and the professional experience of the authors. The literature review identified key themes and gaps related to feedback in EMS, which were further refined through consultation of EMS experts to ensure relevance and clarity. To evaluate comprehensibility and usability, a pretest was conducted with *n* = 26 EMS professionals, whose feedback led to minor modifications before the survey was finalized. For example, information regarding the qualifications of tele-emergency physicians and emergency physicians was consolidated into a single category, i.e., (tele-)emergency physician. Additionally, the number of deployments over a 24-h period instead of 8 h was asked for, as this better reflects the typical shift duration in EMS. The final version of the survey covered general sociodemographic data (*n* = 4 items) along with profession-specific characteristics (*n* = 7 items). The survey explored the following thematic areas:

- **Current state of feedback in EMS (n = 7 items):** This section focused on whether EMS personnel currently receive feedback, how frequently, from whom, what type of feedback, and in which context. Additional questions assessed whether the current feedback mechanisms are perceived as helpful.
- **Assessment of the perceived need of a diagnosis feedback system in EMS (n = 11 items):** This section assessed whether EMS personnel would find feedback regarding their patients’ hospital-confirmed diagnoses helpful for their daily work and would make use of a diagnosis feedback system. The survey also assessed which types of emergency cases (e.g., with or without an emergency physician, specific groups of diagnoses) would particularly benefit from diagnosis feedback, what the preferred maximum time period between the emergency call and the feedback would be, and which EMS professions would benefit the most. Finally, the respondents were asked about further feedback needs.
- **Frameworks for implementing a feedback system in EMS (n = 2 items):** This section addressed elements of the implementation of a feedback system, specifically how feedback can be best provided to EMS staff and whether any existing structures could support its rollout.

Additionally, the survey participants could provide free text comments on the topic of feedback in EMS (referred to as ‘general feedback’, *n* = 1 item). The questionnaire was originally completed by participants in German, and the results were translated for publication. Various question types were applied, including multiple choice questions, Likert scale questions and free text fields.

### Survey distribution

The survey link was distributed via email by the Medical Director of the Communal Rescue Services in the District of Vorpommern-Greifswald to the EMS staff mailing list within the district. In addition, EMS providers in Mecklenburg-Western Pomerania were contacted via publicly available email by the study team with a request to share the survey link with their employees. Two reminder emails were sent through these channels. A third recruitment approach involved the announcement of the survey on social media platforms of the University Medicine Greifswald (Instagram, LinkedIn, X, and Facebook), encouraging participation.

### Data analysis and statistical methods

Questionnaires were included in the analysis if participants met the inclusion criteria and had completed the survey at least up to the questions regarding feedback in EMS. However, apart from the first two questions concerning the eligibility criteria, answering the questions was not mandatory and participants could skip questions.

The results were analyzed descriptively for the total group of respondents (including trainees in EMS professions) and subdivided into the EMS occupational groups (emergency physicians, paramedics, EMTs, and AEMTs) excluding trainees. Participants were subdivided into these EMS groups due to their differing qualifications and respective responsibilities, which may influence their perspectives on feedback in EMS. However, the intention was not to explore group differences. Therefore, no statistical tests were performed. In the case of filter questions, only the frequencies of those who actually answered the question are displayed. Questions allowing multiple responses are indicated accordingly, along with the mean response frequency per participant (indicated as MRF/P), which represents the average number of responses selected by each participant. Categorical variables are presented with absolute and relative frequencies (*n*, %) and numerical variables with the mean and its standard deviation (±SD). Missing values are indicated as separate category. The data were analyzed descriptively using SPSS software (version 29.0).

The free text responses were systematically evaluated and the number of responses was provided. The answers in the free text field for ‘general feedback’ on feedback in EMS were categorized using content analysis [18]. Examples of the categories were selected on the basis of their relevance and comprehensibility, with the aim of presenting a range of perspectives on this topic.

## Results

Of the total *N* = 558 questionnaires, *n* = 130 (23.3%) questionnaires were excluded. Of these, *n* = 9 (1.6%) did not consent to the anonymized use of data, and *n* = 70 (12.5%) were not trained in the EMS sector, resulting in *n* = 79 (14.2%) participants who did not meet the inclusion criteria. Additionally, *n* = 51 (9.1%) questionnaires were not completed up to the questions regarding feedback in EMS. Thus, *N* = 428 questionnaires fulfilled the inclusion criteria and were analyzed respectively.

The survey was completed by *n* = 190 paramedics, *n* = 89 emergency medical technicians (EMTs), *n* = 73 emergency physicians, *n* = 28 advanced emergency medical technicians (AEMTs), *n* = 3 emergency medical responders (EMR), *n* = 1 physician assistant, and *n* = 1 with another, not specified EMS qualification. A further *n* = 43 persons were in training, including *n* = 41 paramedics, n = 1 emergency physician, and *n* = 1 EMT. The results are presented in Tables 1, 2, 3, 4 for all *n* = 428 respondents overall. A more detailed presentation, separated into the four most common EMS professions in Germany with completed training, is provided in Supplementary Tables S1 to S4.

**Table 1:** Sociodemographic and job-related characteristics of the survey participants

	Survey participants N = 428
**Age, mean (±SD)**	36.6 (±13.2)
**Gender, n (%)**	
Male	324 (75.7)
Female	103 (24.1)
Diverse	1 (0.2)
**German federal state with main EMS activity, n (%)**	
Mecklenburg-Western Pomerania	297 (69.4)
Baden-Wuerttemberg	13 (3.0)
Bavaria	9 (2.1)
Berlin	7 (1.6)
Brandenburg	8 (1.9)
Bremen	0 (0)
Hamburg	19 (4.4)
Hesse	13 (3.0)
Lower Saxony	17 (4.0)
North Rhine-Westphalia	23 (5.4)
Rhineland-Palatinate	8 (1.9)
Saarland	0 (0)
Saxony	1 (0.2)
Saxony-Anhalt	1 (0.2)
Schleswig-Holstein	4 (0.9)
Thuringia	8 (1.9)
**Highest qualification in EMS, n (%)**	
Emergency physician	73 (17.1)
Paramedic	190 (44.4)
Advanced emergency medical technician (AEMT)	28 (6.5)
Emergency medical technician (EMT)	89 (20.8)
Emergency medical responder (EMR)	3 (0.7)
I am in training to become an emergency physician	1 (0.2)
I am in training to become a paramedic	41 (9.6)
I am in training to become an AEMT	0 (0)
I am in training to become an EMT	1 (0.2)
I am in training to become an EMR	0 (0)
Other qualification	2 (0.5)
**Duration of employment in the EMS, n (%)**	
Less than 1 year	14 (3.6)
1–5 years	97 (25.2)
6–10 years	86 (22.3)
11–15 years	57 (14.8)
16–20 years	41 (10.6)
More than 20 years	90 (23.4)
**Type of EMS employment, n (%)**	
I work full-time	258 (67.0)
I work part-time, 20 h or more per week	21 (5.5)
I work part-time, less than 20 h per week	25 (6.5)
Only participate in the duty system: regularly	44 (11.4)
Only participate in the duty system: sporadically	26 (6.8)
Other	10 (2.6)
Not answered	1 (0.3)
**Area of responsibility in EMS** **Multiple choice, number of answers (% participants)**	**n = 428 (100%)**
	**571 responses** **1.3 MRF/P**
Emergency rescue (ground ambulance)	404 (94.4)
Emergency rescue (air ambulance)	17 (4.0)
Intensive care transport	34 (7.9)
Qualified patient transport	72 (16.8)
Dispatch center	26 (6.1)
Other	18 (4.2)
**Service area in EMS, n (%)**	
Urban	79 (18.5)
Rural	126 (29.4)
Urban AND rural	222 (51.9)
Not answered	1 (0.2)
**Number of hospitals in the EMS service area, n (%)**	
0–2	113 (26.4)
3–4	179 (41.8)
5–6	63 (14.7)
7–8	32 (7.5)
≥ 9	36 (8.4)
Not answered	5 (1.2)
**Average calls per 24 h, n (%)**	
Fewer than 3 calls	8 (1.9)
3–5 calls	176 (41.1)
6–8 calls	130 (30.4)
More than 8 calls	103 (24.1)
I don’t have calls	7 (1.6)
Not answered	4 (0.9)

**Abbreviations**: MRF/P – mean response frequency per participant

**Table 2:** Current state of feedback in the EMS

	Survey participants N = 428
**Do you currently receive any form of feedback on your work in emergency medical services?** **Multiple choice, number of answers (% participants)**	**n = 428 (100%)**
	**857 responses** **2.0 MRF/P**
No	136 (31.8)
Yes, from colleagues	247 (57.7)
Yes, from supervisors	56 (13.1)
Yes, from patients	127 (29.7)
Yes, from the patients’ relatives	81 (18.9)
Yes, from emergency department staff	130 (30.4)
Yes, from nursing home staff	26 (6.1)
Yes, from the EMS provider (medical director)	42 (9.8)
Other persons	12 (2.8)
*The following two questions were only displayed if participants received some form of feedback on their work*	**N = 292 (100%)**
**In what context do you receive feedback?** **Multiple choice, number of answers (% participants)**	**n = 282 (96.6%)**
	**942 responses** **3.3 MRF/P**
Praise regarding my personal performance	165 (58.5)
Praise regarding team performance	150 (53.2)
Expressions of gratitude	147 (52.1)
Suggestions for improvement regarding my personal performance	94 (33.3)
Suggestions for improvement regarding team performance	75 (26.6)
Debriefing after incidents	215 (76.2)
Complaints	55 (19.5)
Protocol violations/non-compliance with guidelines or SOPs	22 (7.8)
In other contexts	19 (6.7)
**How often do you receive feedback on average (with reference to 2024)? N (%)**	**N = 283 (96.9%)**
Less than once a month	86 (30.4)
Once a month	71 (25.1)
Every two weeks	41 (14.5)
Once a week	39 (13.8)
Several times a week	37 (13.1)
Daily	9 (3.2)
*The following three questions were only displayed if participants received information on the further course of treatment of their patients after transports to the hospital*	**N = 321 (100%)**
**How do you receive information about the further course of treatment of your patients after the transport to the hospital?** **Multiple choice, number of answers (% participants)**	**n = 321 (100%)**
	**474 responses** **1.5 MRF/P**
By asking hospital staff	298 (92.8)
From colleagues	142 (44.2)
From my supervisors	8 (2.5)
By other means	20 (6.2)
Prefer not to answer	6 (1.9)
**What information do you receive?** **Multiple choice, number of answers (% participants)**	**n = 321 (100%)**
	**785 responses** **2.4 MRF/P**
Information on whether the patient survived	281 (87.5)
Admission to inpatient care (yes/no)	134 (41.7)
Primary diagnosis from the hospital	262 (81.6)
Secondary diagnoses from the hospital	60 (18.7)
Medical discharge summary	29 (9.0)
Other information	14 (4.4)
Prefer not to answer	5 (1.6)
**Is the information helpful to you?** **Multiple choice, number of answers (% participants)**	**n = 321 (100%)**
	**1336 responses** **4.2 MRF/P**
Yes, to become more confident in my assessment of the suspected diagnosis	244 (76.0)
Yes, to self-evaluate	248 (77.3)
Yes, to learn for future calls	269 (83.8)
Yes, to feel validated in my work	128 (39.9)
Yes, to gain more motivation for my work because my suspected diagnosis was correct	162 (50.5)
Yes, to better process the calls	129 (40.2)
Yes, to have fewer concerns about possible mistakes after the call	134 (41.7)
The information is helpful for other reasons	15 (4.7)
No	7 (2.2)

*MRF/P* mean response frequency per participant

**Table 3:** Assessment of the need of a diagnosis feedback system in the EMS

	Survey participants N = 428
**Based on your subjective assessment: In how many cases does your suspected diagnosis align with the hospital’s definitive final diagnosis? N (%)**	
≤ 50%	27 (6.3)
50–59%	43 (10.0)
60–69%	49 (11.4)
70–79%	117 (27.3)
80–89%	121 (28.3)
≥ 90%	60 (14.0)
Not answered	11 (2.6)
*The following three questions were only displayed if participants rated the information on the hospital’s final diagnosis as helpful for their daily work in EMS*	**N = 405 (100%)**
**I would like to know the hospital’s final diagnosis in order to…** **Multiple choice, number of answers (% participants)**	**n = 403 (99.5%)**
	**1778 responses** **4.4 MRF/P**
...become more confident in my assessment of the suspected diagnosis	332 (82.4)
...self-evaluate	357 (88.6)
...learn for future calls	382 (94.8)
...feel validated in my work	175 (43.4)
...gain more motivation for my work because my suspected diagnosis was correct	204 (50.6)
...better process the calls	153 (38.0)
...have fewer concerns about possible mistakes after the call	168 (41.7)
...other reasons	7 (1.7)
**For which calls would feedback on the diagnosis be important to you? Multiple choice, number of answers (% participants)**	**n = 399 (98.5%)**
	**894 responses** **2.2 MRF/P**
For calls without an emergency physician	328 (82.2)
For calls with an emergency physician	292 (73.2)
For calls with a tele-emergency physician	175 (43.9)
For specific (suspected) diagnoses: ___	64 (16.0)
For other calls: ____	35 (8.8)
**How soon after a call should diagnostic feedback be provided at the latest? N (%)**	**N = 403 (99.5%)**
As soon as possible	317 (78.7)
Within two weeks	69 (17.1)
Within one month	13 (3.2)
Within three months	0 (0)
Within six months	0 (0)
Within one year	0 (0)
Other time frame	4 (1.0)
**Which professional groups, in your opinion, would benefit from feedback on the diagnosis?** **Multiple choice, number of answers (% participants)**	**n = 427 (99.8%)**
	**675 responses** **1.6 MRF/P**
None	4 (0.9)
All of the mentioned professional groups	335 (78.5)
Emergency physician	73 (17.1)
Tele-emergency physician	40 (9.4)
Paramedic	82 (19.2)
Advanced Emergency Medical Technician (AEMT)	68 (15.9)
Emergency Medical Technician (EMT)	54 (12.6)
Emergency Medical Responder (EMR)	7 (1.6)
Other role	12 (2.8)
**What additional information on the course of treatment would be helpful as feedback after a call for your daily work in EMS?** **Multiple choice, number of answers (% participants)**	**n = 425 (99.3%)**
	**863 responses** **2.0 MRF/P**
None	40 (9.4)
Secondary diagnoses from the hospital	270 (63.5)
Inpatient admission (yes/no)	197 (46.4)
Inpatient stay longer than 24 h (yes/no)	100 (23.5)
Duration of inpatient stay in days	59 (13.9)
Discharge summary including all findings (lab, radiology, etc.)	162 (38.1)
Other information	35 (8.2)

*MRF/P* mean response frequency per participant

**Table 4:** Frameworks for implementing a feedback system in the EMS

	Survey participants N = 428
**What would be the most effective way for you to receive feedback in general? Multiple choice, number of answers (% participants)**	**n = 423 (98.8%)**
	**662 responses** **1.6 MRF/P**
None	3 (0.7)
Directly to me via email	191 (45.2)
Directly to me via mail	14 (3.3)
Through direct digital access	360 (85.1)
Through the EMS provider (medical director)	58 (13.7)
Through other means	36 (8.5)
**In your opinion, which existing structures would be suitable for providing feedback to you or your colleagues?** **Multiple choice, number of answers (% participants)**	**n = 416 (97.2%)**
	**472 responses** **1.1 MRF/P**
None, as I do not wish to receive feedback	5 (1.2)
No existing structure would be suitable	101 (24.3)
As part of post-call debriefing	193 (46.4)
Through a digital system	154 (37.0)
Through another structure	19 (4.6)

*MRF/P* mean response frequency per participant

The socio-demographic characteristics and details of the current employment (job-related information) within the ambulance service are shown in Tables 1 and S1. The mean age of the respondents was 36.6 years (SD ± 13.2 years), with emergency physicians being the oldest group with a mean age of 51.2 years (SD ± 12.3 years). More than 75% (*n* = 324) of the participants were male and almost 70% (*n* = 297) came from Mecklenburg-Western Pomerania. The majority worked full-time in ground-based rescue services (*n* = 404, 94.4%), and nearly 49% (*n* = 188) had been employed in the EMS for over 10 years. Emergency physicians were the group with the longest experience in EMS, with 46.6% (*n* = 34) having worked in EMS for over 20 years, compared to 28.6% (*n* = 8) of AEMTs, 23.7% (*n* = 45) of paramedics, and 3.4% (*n* = 3) of EMTs.

### Current state of feedback in the EMS

A total of 31.8% (*N* = 136) of all respondents stated that they currently do not receive any feedback on their work in the ambulance service (Tables 2 and S2). Among paramedics and AEMTs, the proportion is even higher, at 40.0% and 46.4% respectively. Of those respondents who stated that they receive any feedback, 55.5% (*n* = 157) reported that this occurs on average no more than once a month. When respondents receive feedback, it is mostly from colleagues (57.7%), patients (29.7%), or emergency department staff (30.4%). EMTs receive feedback from colleagues more frequently (72%) than other professional groups (ranging from 39.3% for AEMTs to 61.6% for emergency physicians). In contrast, emergency physicians receive feedback from emergency department staff more often, with nearly 44%, compared to other professional groups (25% among AEMTs to 30% among paramedics). One respondent highlighted the following via the free text fields (*n* = 11 responses):

“*Sometimes, the emergency physician involved calls the hospital for feedback and then passes the information on to the team; in emergency calls without an emergency physician—which is usually the case—this, of course, does not happen.*” [ID = 810, paramedic]

**Fig. 1:**
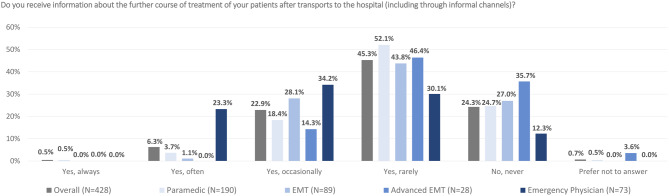
Responses to the question: “Do you receive information about the further course of treatment of your patients after transports to the hospital (including through informal channels)?”

69.6% (*n* = 298) of respondents rarely or never receive information on the further inpatient clinical course of their patients (Fig. 1). Emergency physicians reported receiving feedback more frequently, with 23.3% (*n* = 17) indicating that they often receive feedback about the further course of patient treatment after transport to the hospital. Those respondents, who do, obtain information about the inpatient progress of patients solely through informal feedback requested from hospital staff (92.8%) or colleagues (44.2%). The free-text fields included statements (*n* = 19 responses) such as:

“*I also work in the hospital’s emergency department and check the information myself on the computer*.” (ID = 584, emergency physician)

[I receive feedback…] “*Through informal channels from the emergency physicians involved*.” (ID = 572, paramedic)

Participants report that if so, they mostly receive information about whether the patient survived (87.5%), the final diagnosis from the hospital (81.6%), and whether an inpatient admission was necessary (41.7%, multiple selection question). Most respondents find this information helpful for gaining confidence in assessing the suspected diagnoses (76.0%), for self-evaluation (77.3%), and for learning from it for future calls (83.8%, multiple selection question). Only 7 respondents (2.2%) stated that they do not find the information on their patients’ course of treatment helpful.

### Assessment of the need of a diagnosis feedback system in the EMS

**Fig. 2:**
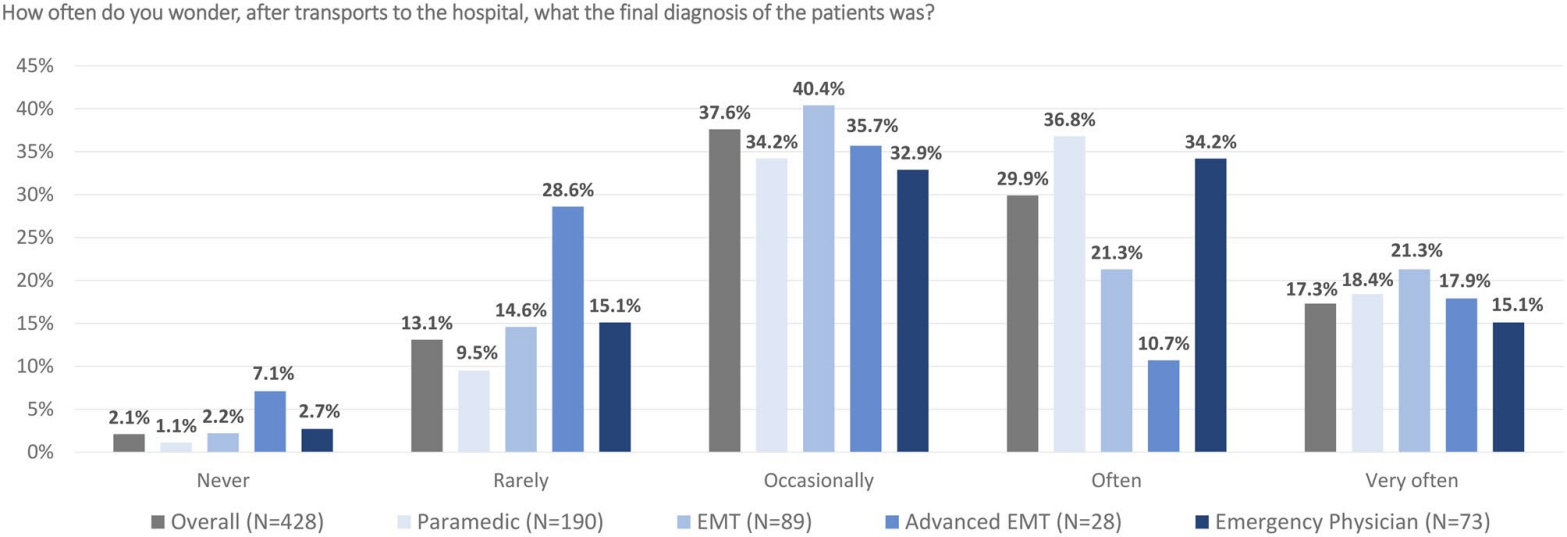
Responses to the question: “How often do you wonder, after transports to the hospital, what the final diagnosis of the patients was?”

**Fig. 3:**
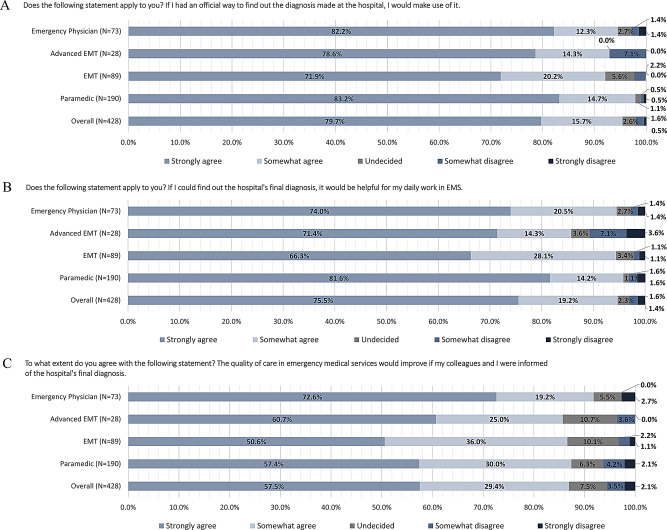
**A** Responses to the question: “Does the following statement apply to you? If I had an official way to find out the diagnosis made at the hospital, I would make use of it.” **B** Responses to the question: “Does the following statement apply to you? If I could find out the hospital’s final diagnosis, it would be helpful for my daily work in EMS.” **C** Responses to the question: “To what extent do you agree with the following statement? The quality of care in emergency medical services would improve if my colleagues and I were informed of the hospital’s final diagnosis”

Of the respondents, 47.2% (*n* = 202) stated that they often or very often ask themselves about the definitive diagnosis of the patients after emergency calls involving hospital transportation (Fig. 2). Thereby, emergency physicians and paramedics stand out in particular, with 49.3% and 55.2%, respectively. Given an official opportunity, 95.4% (*n* = 408) of respondents would take advantage of the opportunity to find out the diagnosis from the hospital (Fig. 3A) and 94.7% (*n* = 405) would perceive this information helpful for their daily work in the EMS (Fig. 3B). In this context, 86.9% (*n* = 372) assume that the quality of patient care would increase if ambulance service staff are given feedback on the final diagnosis of the hospital (Fig. 3C). Further results are shown in Tables 3 and S3. The respondents would like to know the hospitals final diagnosis in order to gain confidence in assessing the suspected diagnoses (82.4%), for self-evaluation (88.6%), and for learning from it for future calls (94.8%, multiple selection question).

The free text field for ‘general feedback’ was used to express:

- the importance of feedback in EMS,
- the error culture,
- the need for further training, knowledge acquisition, and quality improvement,
- aspects of teamwork,
- the type of information required,
- and other topics, such as wishes regarding equipment, specific organizational aspects or feedback on the survey, including appreciation for the initiative.

For example, respondents highlighted the following via the free text fields:

“*There is no better training than to link clinical symptoms and diagnoses with your own emergency deployments. This is an experience for a lifetime. Theoretical training usually only sticks for 14 days.*” (ID = 748, paramedic)

“*I find it very important that action is taken on this issue. In my subjective opinion, the often-lacking feedback is one of the causes of dissatisfaction and lack of error awareness. Feedback is simply essential for improving the quality of EMS operations.*” (ID = 936, paramedic)

“*We are people and we work with people. Separating these people with a wall of data protection and other things is not constructive, especially since, if we try hard enough, we often find out what the patient had, anyway. Making this easier would be a big step forward*.” (ID = 808, paramedic)

*“Feedback and debriefings should be implemented more overall, build greater acceptance of debriefings, improve error culture (e.g. do not look for “culprits”, but instead offer constructive criticism)”* (ID = 1359, emergency physician)

### Frameworks for implementing a feedback system in the EMS

The respondents would mostly prefer to receive feedback via direct digital access (*n* = 360, 85.1%) or by email (*n* = 191, 45.2%). Feedback could be provided as part of the debriefing (*n* = 193, 46.4%) or via an existing digital system (*n* = 154, 37.0%). For more details, see Tables 4 and S4.

## Discussion

We present the results from the first comprehensive survey on feedback practice and preference among emergency ambulance staff in Germany, encompassing responses from over 400 participants. We found that one out of three EMS employees does not receive any feedback on her or his work, and more than half of those who receive feedback only receive it once per month or less frequently. Additionally, a quarter of respondents never receive information about patients’ subsequent treatment after transportation to the hospital. When EMS staff receive feedback, it is typically through personal inquiries at the hospital or informal updates from colleagues. Nearly 48% of respondents wonder often to very often what the inpatient diagnosis of their patients is. This information would help them in their daily work in the ambulance service and enable them to learn for future cases. More than 90% would like to receive official feedback on the hospital diagnosis, and similarly, 85% state that diagnosis feedback would improve the quality of ambulance service care.

In 2022, Keimer et al. conducted qualitative interviews with six EMS professionals in Germany regarding the need for a digital feedback system [19]. In line with our study, the interviews revealed that EMS professionals rarely or never receive feedback regarding the patient’s diagnosis or well-being. Keimer and colleagues concluded that an automated feedback system is urgently needed to improve quality of care, patient outcomes, and communication between hospitals and EMS personnel [19]. In the city of Braunschweig, a feedback system was implemented in 2017 which provides EMS staff and dispatchers post-resuscitation patient outcome feedback for quality assurance purposes [20, 21]. The EMS professionals receive feedback on whether the patient was discharged from hospital alive and what the outcome was at discharge with regard to the neurological status (cerebral performance category) [22]. Günther et al. reported that in 2018, the system was expanded to notify EMS personnel of patient deaths occurring within one day of ambulance contact when no emergency physician was involved [20]. As mentioned by the authors, such feedback systems are fundamental for professional and personal development, offering opportunities for reflection, motivation, and ultimately enhancing patient safety. However, this feedback system is limited to patients who are already in need of resuscitation or have died, patients for whom the EMS personnel’s scope of action was limited anyway. The survey showed that feedback on unclear medical conditions (such as dyspnea) would be more relevant, e.g., to know whether the patient was taken to the appropriate hospital.

To the best of our knowledge, the above described system is currently the only structured patient outcome feedback system for non-physician EMS personnel in Germany. This conclusion is supported by Klausen et al., who conducted a systematic literature review on cross-sectoral digital feedback systems in EMS, identifying only Günther et al.’s study (2018) as meeting the inclusion criteria [23]. Today, evidence is available that post-resuscitation feedback improves the quality of resuscitation [24]. The German Resuscitation Registry (GRR) could serve as a valuable cross-sectoral data source, though its data primarily pertains to cardiac arrest and resuscitation cases and does not benefit the EMS personnel involved directly [25].

The lack of feedback in emergency medical services seems to be an internationally relevant topic, as qualitative studies from the UK [26] and Canada [27] have also highlighted that EMS professionals have a strong desire for feedback and participants viewed current feedback provision as inadequate. Morrison et al. highlighted that the adaptive development of informal feedback structures suggests a lack of a structured, adequate feedback system [27]. Additionally, Wilson et al. developed a logic model for prehospital feedback interventions, outlining a structured approach to enhance future research [26]. The study identified potential individual psychological processes and outcomes resulting from feedback in EMS, including effects on motivation, job satisfaction, behavioral changes, learning, and closure. Additionally, Wilson et al. highlighted organizational outcomes for EMS, divided into workforce outcomes, such as improved mental health of staff, enhanced engagement, and a better work environment, and core quality outcomes, such as improved clinical performance, increased patient safety, and higher service quality. Interestingly, Wilson et al. found that improving patient care was only mentioned in connection with patient outcome feedback, whilst patient-experience feedback was perceived as only being associated with desiring reassurance and praise [26]. A systematic review and meta-analysis on types and effects of feedback for emergency ambulance staff concluded that feedback was found to have a moderate positive effect on quality of care and professional development. However, interventions within EMS that explore patient outcome feedback are needed as they were desired by staff and are currently under-represented in interventional studies [15].

In Germany, the federal states are responsible for quality assurance in EMS. An exploration of the federal state laws reveals that there are hardly any uniform standards; the data protection frameworks, responsibilities and requirements differ considerably. With regard to quality assurance, Baden-Wuerttemberg can be considered a model federal state with a central office for cross-provider quality assurance (SQR-BW) [16, 28]. This office has established a centralized online portal (SQR-BW-Portal) to manage data from service providers, on-site emergency medical management teams, integrated control centers, and tele-emergency medical control centers. By law, the SQR-BW is authorized to collect, link, store, adapt, retrieve, and modify this data [29]. Additionally, the SQR-BW is permitted to transmit data to external persons or offices when necessary, such as for the further development of the ambulance service, quality assurance measures, or for scientific and research purposes [29]. Further federal states with a central office for quality assurance are Schleswig-Holstein, Hamburg and Rhineland-Palatinate. The other federal states delegate responsibility for quality assurance to the medical director of the rescue service or the responsible ministries themselves are responsible for quality assurance measures. Therefore, no significant improvement is to be expected with regard to standardized feedback systems for EMS personnel as a result of the current reforms. In this regard, action is urgently needed. This study showed that non-physician personnel in particular receive less feedback on the further inpatient treatment course, which, in the context of staff shortages and the expansion of the competencies of paramedics, can lead to quality gaps, which are currently emerging in Germany [3].

### Limitations and strengths

This study has certain limitations. First, the results may not be representative for all of German EMS personnel. Due to the different EMS providers, no official statistics are available on the total number of EMS staff in Germany. Therefore, it was not possible to determine the response rate. Due to Germany’s federal structures, the survey primarily targeted Mecklenburg-Western Pomerania, which further limits the generalizability of the survey results. Second, there is a risk of self-selection bias, as the survey was voluntary and anonymous. It can be assumed that people who are interested in the topic were more likely to participate and thus the results might not be fully representative. Due to the anonymous nature of the survey, it is possible that some participants initially opened the questionnaire but discontinued it, only to re-open and complete it at a later time. This may have contributed to the considerably high number of excluded questionnaires. Nevertheless, this was the first comprehensive survey with over 400 EMS employees on the topic of feedback, and hardly any missing values were detected in the questionnaires included. The feedback to the survey was very positive, i.e., the participants emphasized the importance of the issue. Moreover, the survey was conducted anonymously, which allows respondents to express their opinions without fear of consequences. This anonymity has been shown to engage higher levels of self-disclosure [30]. For this reason, it can be assumed that the statements reflect the wishes and concerns of EMS staff.

## Conclusion

While structured feedback on patients’ diagnoses and outcomes is strongly desired by EMS staff and could improve the quality of patient care and personnel professional development, significant infrastructural, legal and administrative barriers currently hinder the implementation of digital feedback systems. The federalized EMS structures in Germany, with its intersectoral and interdisciplinary patient care, have so far prevented the development of harmonized, standardized, consistent and ideally digital quality management and assurance frameworks. Given the expanding responsibilities of non-physician EMS personnel and the growing frequency of cases without physician involvement, the need for such digital feedback structures is even intensifying.

Within the framework of a pilot study, the benefits of a digital diagnosis feedback system should be evaluated in terms of improving the quality of care and strengthening self-reflection of EMS staff. Importantly, the data protection requirements of the German federal states for the use of EMS data for quality assurance and research purposes should be harmonized.

## Electronic supplementary material

Below is the link to the electronic supplementary material.

Supplementary Material 1

## Data Availability

All data collected are presented descriptively within the manuscript. Data usage requests for further analyzes can be submitted to the corresponding author.
