# Supplementary material for: The urgent need for patients’ diagnoses and outcome feedback in Germany’s emergency medical services — insights from a web-based survey
Source: BMC Emerg Med. 2025 Apr 20;25:66. doi: 10.1186/s12873-025-01218-8 (PMC12010660; doi:10.1186/s12873-025-01218-8)
Supplement: Supplementary file 1 — Supplementary Material 1 [file 12873_2025_1218_MOESM1_ESM.docx]

**Supplementary Table S1** – Sociodemographic and job-related characteristics of the survey participants stratified by occupational groups

|  | **Emergency physician**  **N=73** | **Paramedic**  **N=190** | **AEMT**  **N=28** | **EMT**  **N=89** |
| --- | --- | --- | --- | --- |
| **Age, mean (±SD)** | 51.2 (±12.3) | 36.5 (±10.0) | 43.5 (±10.3) | 29.2 (±10.6) |
| **Gender, n (%)**  male  female  diverse | 50 (68.5)  23 (31.5)  0 (0) | 151 (79.5)  39 (20.5)  0 (0) | 24 (85.7)  4 (14.3)  0 (0) | 65 (73.0)  24 (27.0)  0 (0) |
| **German federal state with main EMS activity, n (%)**  Mecklenburg-Western Pomerania  Baden-Wuerttemberg  Bavaria  Berlin  Brandenburg  Bremen  Hamburg  Hesse  Lower Saxony  North Rhine-Westphalia  Rhineland-Palatinate  Saarland  Saxony  Saxony-Anhalt  Schleswig-Holstein  Thuringia | 59 (80.8)  0 (0)  0 (0)  5 (6.8)  2 (2.7)  0 (0)  2 (2.7)  0 (0)  1 (1.4)  0 (0)  1 (1.4)  0 (0)  0 (0)  1 (1.4)  0 (0)  2 (2.7) | 135 (71.1)  9 (4.7)  1 (0.5)  1 (0.5)  2 (1.1)  0 (0)  7 (3.7)  2 (1.1)  8 (4.2)  14 (7.4)  4 (2.1)  0 (0)  0 (0)  0 (0)  3 (1.6)  4 (2.1) | 18 (64.3)  1 (3.6)  1 (3.6)  0 (0)  0 (0)  0 (0)  1 (3.6)  1 (3.6)  2 (7.1)  3 (10.7)  0 (0)  0 (0)  0 (0)  0 (0)  0 (0)  1 (3.6) | 52 (58.4)  2 (2.2)  6 (6.7)  1 (1.1)  2 (2.2)  0 (0)  7 (7.9)  5 (5.6)  6 (6.7)  4 (4.5)  3 (3.4)  0 (0)  1 (1.1)  0 (0)  0 (0)  0 (0) |
| **Duration of employment in the EMS, n (%)**  Less than 1 year  1-5 years  6-10 years  11-15 years  16-20 years  More than 20 years | 0 (0)  13 (17.8)  14 (19.2)  6 (8.2)  6 (8.2)  34 (46.6) | 2 (1.1)  35 (18.4)  45 (23.7)  38 (20.0)  25 (13.2)  45 (23.7) | 0 (0)  2 (7.1)  6 (21.4)  6 (21.4)  6 (21.4)  8 (28.6) | 11 (12.4)  45 (50.6)  20 (22.5)  6 (6.7)  4 (4.5)  3 (3.4) |
| **Type of EMS employment, n (%)**  I work full-time  I work part-time, 20 hours or more per week  I work part-time, less than 20 hours per week  Only participate in the duty system: regularly  Only participate in the duty system: sporadically  Other  Not answered | 17 (23.3)  8 (11.0)  4 (5.5)  36 (49.3)  4 (5.5)  4 (5.5)  0 (0) | 160 (84.2)  7 (3.7)  9 (4.7)  3 (1.6)  7 (3.7)  4 (2.1)  0 (0) | 14 (50.0)  2 (7.1)  3 (10.7)  3 (10.7)  6 (21.4)  0 (0)  0 (0) | 64 (71.9)  3 (3.4)  9 (10.1)  2 (2.2)  8 (9.0)  2 (2.2)  1 (1.1) |
| **Area of responsibility in EMS**  **Multiple choice, number of answers (% participants)** | **n=73 (100%)** | **n=190 (100%)** | **n=28 (100%)** | **n=89 (100%)** |
|  | **102 responses**  **1.4 MRF/P** | **243 responses**  **1.2 MRF/P** | **35 responses**  **1.3 MRF/P** | **130 responses**  **1.5 MRF/P** |
| Emergency rescue (ground ambulance)  Emergency rescue (air ambulance)  Intensive care transport  Qualified patient transport  Dispatch center  Other | 70 (95.9)  11 (15.1)  16 (21.9)  1 (1.4)  0 (0)  4 (5.5) | 184 (96.8)  5 (2.6)  8 (4.2)  20 (10.5)  17 (8.9)  9 (4.7) | 22 (78.6)  0 (0)  0 (0)  3 (10.7)  8 (28.6)  2 (7.1) | 83 (93.3)  0 (0)  8 (9.0)  38 (42.7)  0 (0)  1 (1.1) |
| **Service area in EMS, n (%)**  Urban  Rural  Urban AND Rural  Not Answered | 7 (9.6)  18 (24.7)  48 (65.8)  0 (0) | 34 (17.9)  58 (30.5)  98 (51.6)  0 (0) | 5 (17.9)  8 (28.6)  14 (50.0)  1 (3.6) | 21 (23.6)  23 (25.8)  45 (50.6)  0 (0) |
| **Number of hospitals in the EMS service area, n (%)**  0 to 2  3 to 4  5 to 6  7 to 8  ≥ 9  Not Answered | 6 (8.2)  33 (45.2)  13 (17.8)  7 (9.6)  11 (15.1)  3 (4.1) | 55 (28.9)  84 (44.2)  26 (13.7)  14 (7.4)  9 (4.7)  2 (1.1) | 9 (32.1)  9 (32.1)  7 (25.0)  2 (7.1)  1 (3.6)  0 (0) | 26 (29.2)  33 (37.1)  11 (12.4)  8 (9.0)  11 (12.4)  0 (0) |
| **Average calls per 24 hours, n (%)**  Fewer than 3 calls  3 to 5 calls  6 to 8 calls  More than 8 calls  I don’t have calls  Not Answered | 4 (5.5)  43 (58.9)  12 (16.4)  14 (19.2)  0 (0)  0 (0) | 2 (1.1)  68 (35.8)  66 (34.7)  50 (26.3)  3 (1.6)  1 (0.5) | 1 (3.6)  7 (25.0)  8 (28.6)  7 (25.0)  4 (14.3)  1 (3.6) | 1 (1.1)  35 (39.3)  32 (36.0)  20 (22.5)  0 (0)  1 (1.1) |

**Abbreviations**: AEMT – Advanced Emergency Medical Technician; EMT – Emergency Medical Technician; MRF/P – mean response frequency per participant

**Supplementary** T**able S2** – Current state of feedback in the EMS – results stratified by occupational groups

|  | **Emergency physician**  **N=73** | **Paramedic**  **N=190** | **AEMT**  **N=28** | **EMT**  **N=89** |
| --- | --- | --- | --- | --- |
| **Do you currently receive any form of feedback on your work in emergency medical services?**  **Multiple choice, number of answers (% participants)** | **n=73 (100%)** | **n=190 (100%)** | **n=28 (100%)** | **n=89 (100%)** |
|  | **135 responses**  **1.8 MRF/P** | **379 responses**  **2.0 MRF/P** | **53 responses**  **1.9 MRF/P** | **192 responses**  **2.2 MRF/P** |
| No  Yes, from colleagues  Yes, from supervisors  Yes, from patients  Yes, from the patients’ relatives  Yes, from emergency department staff  Yes, from nursing home staff  Yes, from the EMS provider (medical director)  Other persons | 19 (26.0)  45 (61.6)  4 (5.5)  11 (15.1)  7 (9.6)  32 (43.8)  5 (6.8)  11 (15.1)  1 (1.4) | 76 (40.0)  91 (47.9)  22 (11.6)  58 (30.5)  41 (21.6)  56 (29.5)  10 (5.3)  19 (10.0)  6 (3.2) | 13 (46.4)  11 (39.3)  4 (14.3)  7 (25.0)  6 (21.4)  7 (25.0)  1 (3.6)  3 (10.7)  1 (3.6) | 19 (21.3)  64 (71.9)  16 (18.0)  36 (40.4)  20 (22.5)  23 (25.8)  7 (7.9)  6 (6.7)  1 (1.1) |
| *Questions shaded in gray were only displayed if participants received some form of feedback on their work.* | **N=54 (100%)** | **N=114 (100%)** | **N=15 (100%)** | **N=70 (100%)** |
| **In what context do you receive feedback?**  **Multiple choice, number of answers (% participants)** | **n=53 (98.1%)** | **n=109 (95.6%)** | **n=14 (93.3%)** | **n=68 (97.1%)** |
|  | **154 responses**  **2.9 MRF/P** | **343 responses**  **3.1 MRF/P** | **42 responses**  **3.0 MRF/P** | **248 responses**  **3.6 MRF/P** |
| Praise regarding my personal performance  Praise regarding team performance  Expressions of gratitude  Suggestions for improvement regarding my personal performance  Suggestions for improvement regarding team performance  Debriefing after incidents  Complaints  Protocol violations/non-compliance with guidelines or SOPs  In other contexts | 20 (37.7)  25 (47.2)  16 (30.2)  10 (18.9)  13 (24.5)  42 (79.2)  12 (22.6)  8 (15.1)  8 (15.1) | 57 (52.3)  54 (49.5)  68 (62.4)  22 (20.2)  23 (21.1)  80 (73.4)  22 (20.2)  10 (9.2)  7 (6.4) | 8 (57.1)  6 (42.9)  8 (57.1)  4 (28.6)  3 (21.4)  9 (64.3)  4 (28.6)  0 (0)  0 (0) | 52 (76.5)  43 (63.2)  35 (51.5)  31 (45.6)  21 (30.9)  49 (72.1)  13 (19.1)  2 (2.9)  2 (2.9) |
| **How often do you receive feedback on average (with reference to 2024)? N (%)** | **N=53 (98.1%)** | **N=110 (96.5%)** | **N=13 (86.7%)** | **N=68 (97.1%)** |
| Less than once a month  Once a month  Every two weeks  Once a week  Several times a week  Daily | 18 (34.0)  22 (41.5)  5 (9.4)  2 (3.8)  4 (7.5)  2 (3.8) | 40 (36.4)  27 (24.5)  19 (17.3)  11 (10.0)  12 (10.9)  1 (0.9) | 9 (69.2)  1 (7.7)  2 (15.4)  0 (0)  1 (7.7)  0 (0) | 16 (23.5)  15 (22.1)  7 (10.3)  18 (26.5)  10 (14.7)  2 (2.9) |
| *Questions shaded in gray were only displayed if participants received information on the further course of treatment of their patients after transports to the hospital.* | **N=64 (100%)** | **N=142 (100%)** | **N=17 (100%)** | **N=65 (100%)** |
| **How do you receive information about the further course of treatment of your patients after the transport to the hospital?**  **Multiple choice, number of answers (% participants)** | **n=64 (100%)** | **n=142 (100%)** | **n=17 (100%)** | **n=65 (100%)** |
|  | **104 responses**  **1.6 MRF/P** | **208 responses**  **1.5 MRF/P** | **20 responses**  **1.2 MRF/P** | **94 responses**  **1.5 MRF/P** |
| By asking hospital staff  From colleagues  From my supervisors  By other means  Prefer not to answer | 56 (87.5)  34 (53.1)  3 (4.7)  10 (15.6)  1 (1.6) | 138 (97.2)  57 (40.1)  5 (3.5)  7 (4.9)  1 (0.7) | 15 (88.2)  3 (17.6)  0 (0)  0 (0)  2 (11.8) | 60 (92.3)  31 (47.7)  0 (0)  1 (1.5)  2 (2.1) |
| **What information do you receive?**  **Multiple choice, number of answers (% participants)** | **n=64 (100%)** | **n=142 (100%)** | **n=17 (100%)** | **n=65 (100%)** |
|  | **173 responses**  **2.7 MRF/P** | **347 responses**  **2.4 MRF/P** | **32 responses**  **1.9 MRF/P** | **145 responses**  **2.2 MRF/P** |
| Information on whether the patient survived  Admission to inpatient care (yes/no)  Primary diagnosis from the hospital  Secondary diagnoses from the hospital  Medical discharge summary  Other information  Prefer not to answer | 56 (87.5)  30 (46.9)  56 (87.5)  15 (23.4)  12 (18.8)  4 (6.3)  0 (0) | 122 (85.9)  62 (43.7)  113 (79.6)  27 (19.0)  12 (8.5)  8 (5.6)  3 (2.1) | 14 (82.4)  6 (35.3)  10 (58.8)  1 (5.9)  0 (0)  0 (0)  1 (5.9) | 59 (90.8)  18 (27.7)  54 (83.1)  9 (13.8)  3 (4.6)  1 (1.5)  1 (1.5) |
| **Is the information helpful to you?**  **Multiple choice, number of answers (% participants)** | **n=64 (100%)** | **n=142 (100%)** | **n=17 (100%)** | **n=65 (100%)** |
|  | **225 responses**  **3.5 MRF/P** | **634 responses**  **4.5 MRF/P** | **61 responses**  **3.6 MRF/P** | **283 responses**  **4.4 MRF/P** |
| Yes, to become more confident in my assessment of the suspected diagnosis  Yes, to self-evaluate  Yes, to learn for future calls  Yes, to feel validated in my work  Yes, to gain more motivation for my work because my suspected diagnosis was correct  Yes, to better process the calls  Yes, to have fewer concerns about possible mistakes after the call  The information is helpful for other reasons  No | 36 (56.3)  52 (81.3)  52 (81.3)  17 (26.6)  20 (31.3)  21 (32.8)  18 (28.1)  5 (7.8)  4 (6.3) | 115 (81.0)  115 (81.0)  121 (85.2)  68 (47.9)  85 (59.9)  56 (39.4)  64 (45.1)  8 (5.6)  2 (1.4) | 13 (76.5)  9 (52.9)  14 (82.4)  4 (23.5)  9 (52.9)  5 (29.4)  7 (41.2)  0 (0)  0 (0) | 53 (81.5)  49 (75.4)  57 (87.7)  27 (41.5)  33 (50.8)  32 (49.2)  31 (47.7)  1 (1.5)  0 (0) |

**Abbreviations**: AEMT – Advanced Emergency Medical Technician; EMT – Emergency Medical Technician; MRF/P – mean response frequency per participant

**Supplementary Table S3** – Assessment of the need of a diagnosis feedback system in the EMS – results stratified by occupational groups

|  | **Emergency physician**  **N=73** | **Paramedic**  **N=190** | **AEMT**  **N=28** | **EMT**  **N=89** |
| --- | --- | --- | --- | --- |
| **Based on your subjective assessment: In how many cases does your suspected diagnosis align with the hospital’s definitive final diagnosis? N (%)**  ≤ 50%  50 % to 59 %  60 % to 69 %  70 % to 79 %  80 % to 89 %  ≥ 90 %  Not answered | 5 (6.8)  6 (8.2)  7 (9.6)  21 (28.8)  24 (32.9)  8 (11.0)  2 (2.7) | 7 (3.7)  16 (8.4)  27 (14.2)  50 (26.3)  58 (30.5)  28 (14.7)  4 (2.1) | 2 (7.1)  4 (14.3)  1 (3.6)  10 (35.7)  2 (7.1)  7 (25.0)  2 (7.1) | 5 (5.6)  12 (13.5)  9 (10.1)  28 (31.5)  21 (23.6)  12 (13.5)  2 (2.2) |
| *Questions shaded in gray were only displayed if participants rated the information on the hospital's final diagnosis as helpful for their daily work in EMS.* | **N=69 (100%)** | **N=182 (100%)** | **N=24 (100%)** | **N=84 (100%)** |
| **I would like to know the hospital's final diagnosis in order to ...**  **Multiple choice, number of answers (% participants)** | **n=68 (98.6%)** | **n=182 (100%)** | **n=24 (100%)** | **n=83 (98.8%)** |
|  | **256 responses**  **3.8 MRF/P** | **842 responses**  **4.6 MRF/P** | **103 responses**  **4.3 MRF/P** | **386 responses**  **4.7 MRF/P** |
| ... become more confident in my assessment of the suspected  diagnosis  ... self-evaluate  ... learn for future calls  ... feel validated in my work  ... gain more motivation for my work because my suspected  diagnosis was correct  ... better process the calls  ... have fewer concerns about possible mistakes after the call  ... other reasons | 39 (57.4)  61 (89.7)  65 (95.6)  25 (36.8)  18 (26.5)  26 (38.2)  21 (30.9)  1 (1.5) | 160 (87.9)  170 (93.4)  173 (95.1)  89 (48.9)  108 (59.3)  63 (34.6)  78 (42.9)  1 (0.5) | 20 (83.3)  19 (79.2)  23 (95.8)  9 (37.5)  11 (45.8)  9 (37.5)  10 (41.7)  2 (8.3) | 72 (86.7)  72 (86.7)  78 (94.0)  37 (44.6)  46 (55.4)  39 (47.0)  40 (48.2)  2 (2.4) |
| **For which calls would feedback on the diagnosis be important to you? Multiple choice, number of answers (% participants)** | **n=66 (95.7%)** | **n=182 (100%)** | **n=24 (100%)** | **n=81 (96.4%)** |
|  | **102 responses**  **1.5 MRF/P** | **445 responses**  **2.4 MRF/P** | **54 responses**  **2.3 MRF/P** | **199 responses**  **2.5 MRF/P** |
| For calls without an emergency physician  For calls with an emergency physician  For calls with a tele-emergency physician  For specific (suspected) diagnoses: ___  For other calls: ____ | 12 (18.2)  51 (77.3)  12 (18.2)  14 (21.2)  13 (19.7) | 171 (94.0)  135 (74.2)  90 (49.5)  32 (17.6)  17 (9.3) | 23 (95.8)  17 (70.8)  10 (41.7)  2 (8.3)  2 (8.3) | 79 (97.5)  57 (70.4)  47 (58.0)  14 (17.3  2 (2.5) |
| **How soon after a call should diagnostic feedback be provided at the latest? N (%)** | **N=68 (98.6%)** | **N=182 (100%)** | **N=24 (100%)** | **N=83 (98.8%)** |
| As soon as possible  Within two weeks  Within one month  Within three months  Within six months  Within one year  Other time frame | 45 (66.2)  19 (27.9)  4 (5.9)  0 (0)  0 (0)  0 (0)  0 (0) | 146 (80.2)  30 (16.5)  4 (2.2)  0 (0)  0 (0)  0 (0)  2 (1.1) | 20 (83.3)  3 (12.5)  1 (4.2)  0 (0)  0 (0)  0 (0)  0 (0) | 67 (80.7)  10 (12.0)  4 (4.8)  0 (0)  0 (0)  0 (0)  2 (2.4) |
| **Which professional groups, in your opinion, would benefit from feedback on the diagnosis?**  **Multiple choice, number of answers (% participants)** | **n=73 (100%)** | **n=189 (99.5%)** | **n=28 (100%)** | **n=89 (100%)** |
|  | **104 responses**  **1.4 MRF/P** | **323 responses**  **1.7 MRF/P** | **35 responses**  **1.3 MRF/P** | **136 responses**  **1.5 MRF/P** |
| None  All of the mentioned professional groups  Emergency physician  Tele-emergency physician  Paramedic  Advanced Emergency Medical Technician (AEMT)  Emergency Medical Technician (EMT)  Emergency Medical Responder (EMR)  Other role | 2 (2.7)  59 (80.8)  11 (15.1)  5 (6.8)  10 (13.7)  7 (9.6)  7 (9.6)  1 (1.4)  2 (2.7) | 2 (1.1)  145 (76.7)  36 (19.0)  25 (13.2)  41 (21.7)  37 (19.6)  25 (13.2)  5 (2.6)  7 (3.7) | 0 (0)  24 (85.7)  3 (10.7)  2 (7.1)  2 (7.1)  2 (7.1)  1 (3.6)  0 (0)  1 (3.6) | 0 (0)  72 (80.9)  13 (14.6)  4 (4.5)  16 (18.0)  13 (14.6)  15 (16.9)  1 (1.1)  2 (2.2) |
| **What additional information on the course of treatment would be helpful as feedback after a call for your daily work in EMS?**  **Multiple choice, number of answers (% participants)** | **n=73 (100%)** | **n=188 (98.9%)** | **n=28 (100%)** | **n=89 (100%)** |
|  | **128 responses**  **1.8 MRF/P** | **404 responses**  **2.1 MRF/P** | **51 responses**  **1.8 MRF/P** | **192 responses**  **2.2 MRF/P** |
| None  Secondary diagnoses from the hospital  Inpatient admission (yes/no)  Inpatient stay longer than 24 hours (yes/no)  Duration of inpatient stay in days  Discharge summary including all findings (lab, radiology, etc.)  Other information | 7 (9.6)  28 (38.4)  32 (43.8)  10 (13.7)  7 (9.6)  37 (50.7)  7 (9.6) | 18 (9.6)  131 (69.7)  99 (52.7)  48 (25.5)  26 (13.8)  65 (34.6)  17 (9.0) | 3 (10.7)  19 (67.9)  10 (35.7)  6 (21.4)  3 (10.7)  5 (17.9)  5 (17.9) | 11 (12.4)  60 (67.4)  38 (42.7)  26 (29.2)  18 (20.2)  37 (41.6)  2 (2.2) |

**Abbreviations**: AEMT – Advanced Emergency Medical Technician; EMT – Emergency Medical Technician; MRF/P – mean response frequency per participant

**Supplementary Table S4** – Frameworks for implementing a feedback system in the EMS – results stratified by occupational groups

|  | **Emergency physician**  **N=73** | **Paramedic**  **N=190** | **AEMT**  **N=28** | **EMT**  **N=89** |
| --- | --- | --- | --- | --- |
| **What would be the most effective way for you to receive feedback in general? Multiple choice, number of answers (% participants)** | **n=72 (98.6%)** | **n=188 (98.9%)** | **n=28 (100%)** | **n=87 (97.8%)** |
|  | **119 responses**  **1.7 MRF/P** | **288 responses**  **1.5 MRF/P** | **41 responses**  **1.5 MRF/P** | **136 responses**  **1.6 MRF/P** |
| None  Directly to me via email  Directly to me via mail  Through direct digital access  Through the EMS provider (medical director)  Through other means | 1 (1.4)  44 (61.1)  3 (4.2)  54 (75.0)  9 (12.5)  8 (11.1) | 1 (0.5)  83 (44.1)  3 (1.6)  159 (84.6)  25 (13.3)  17 (9.0) | 0 (0)  8 (28.6)  0 (0)  25 (89.3)  6 (21.4)  2 (7.1) | 0 (0)  37 (42.5)  3 (3.4)  79 (90.8)  11 (12.6)  6 (6.9) |
| **In your opinion, which existing structures would be suitable for providing feedback to you or your colleagues?**  **Multiple choice, total responses (% participants)** | **n=72 (98.6%)** | **n=184 (96.8%)** | **n=26 (92.9%)** | **n=86 (96.6%)** |
|  | **85 responses**  **1.2 MRF/P** | **210 responses**  **1.1 MRF/P** | **28 responses**  **1.1 MRF/P** | **96 responses**  **1.2 MRF/P** |
| None, as I do not wish to receive feedback  No existing structure would be suitable  As part of post-call debriefing  Through a digital system  Through another structure | 2 (2.8)  15 (20.8)  27 (37.5)  33 (45.8)  8 (11.1) | 2 (1.1)  39 (21.2)  80 (43.5)  78 (42.4)  11 (6.0) | 0 (0)  10 (38.5)  9 (34.6)  9 (34.6)  0 (0) | 0 (0)  22 (25.6)  47 (54.7)  27 (31.4)  0 (0) |

**Abbreviations**: AEMT – Advanced Emergency Medical Technician; EMT – Emergency Medical Technician; MRF/P – mean response frequency per participant
